# D-dimer as a biomarker for disease severity and mortality in COVID-19 patients: a case control study

**DOI:** 10.1186/s40560-020-00466-z

**Published:** 2020-07-10

**Authors:** Yumeng Yao, Jiatian Cao, Qingqing Wang, Qingfeng Shi, Kai Liu, Zhe Luo, Xiang Chen, Sisi Chen, Kaihuan Yu, Zheyong Huang, Bijie Hu

**Affiliations:** 1 Department of Infectious Diseases, Zhongshan Hospital, Fudan University, 180 Feng Lin Road, Shanghai, 200032 China; 2 Shanghai Institute of Cardiovascular Diseases, Zhongshan Hospital, Fudan University, 180 Feng Lin Road, Shanghai, 200032 China; 3 Department of Infection Control, Zhongshan Hospital, Fudan University, 180 Feng Lin Road, Shanghai, 200032 China; 4 Department of Critical Care Medicine, Zhongshan Hospital, Fudan University, 180 Feng Lin Road, Shanghai, 200032 China; 5 Department of Cardiology, Renmin Hospital, Wuhan University, Gaoxin 6th Road, Donghu High Tech Development Zone, Wuhan City, 430200 China; 6 Department of Hepatobiliary Endoscopic Surgery, Renmin Hospital, Wuhan University, Gaoxin 6th Road, Donghu High Tech Development Zone, Wuhan City, 430200 China

**Keywords:** D-dimer, Coronavirus disease-19, Biomarker, Severity, Mortality

## Abstract

**Background:**

Over 5,488,000 cases of coronavirus disease-19 (COVID-19) have been reported since December 2019. We aim to explore risk factors associated with mortality in COVID-19 patients and assess the use of D-dimer as a biomarker for disease severity and clinical outcome.

**Methods:**

We retrospectively analyzed the clinical, laboratory, and radiological characteristics of 248 consecutive cases of COVID-19 in Renmin Hospital of Wuhan University, Wuhan, China from January 28 to March 08, 2020. Univariable and multivariable logistic regression methods were used to explore risk factors associated with in-hospital mortality. Correlations of D-dimer upon admission with disease severity and in-hospital mortality were analyzed. Receiver operating characteristic curve was used to determine the optimal cutoff level for D-dimer that discriminated those survivors versus non-survivors during hospitalization.

**Results:**

Multivariable regression that showed D-dimer > 2.0 mg/L at admission was the only variable associated with increased odds of mortality [OR 10.17 (95% CI 1.10–94.38), *P* = 0.041]. D-dimer elevation (≥ 0.50 mg/L) was seen in 74.6% (185/248) of the patients. Pulmonary embolism and deep vein thrombosis were ruled out in patients with high probability of thrombosis. D-dimer levels significantly increased with increasing severity of COVID-19 as determined by clinical staging (Kendall’s tau-b = 0.374, *P* = 0.000) and chest CT staging (Kendall’s tau-b = 0.378, *P* = 0.000). In-hospital mortality rate was 6.9%. Median D-dimer level in non-survivors (*n* = 17) was significantly higher than in survivors (*n* = 231) [6.21 (3.79–16.01) mg/L versus 1.02 (0.47–2.66) mg/L, *P* = 0.000]. D-dimer level of > 2.14 mg/L predicted in-hospital mortality with a sensitivity of 88.2% and specificity of 71.3% (AUC 0.85; 95% CI = 0.77–0.92).

**Conclusions:**

D-dimer is commonly elevated in patients with COVID-19. D-dimer levels correlate with disease severity and are a reliable prognostic marker for in-hospital mortality in patients admitted for COVID-19.

## Background

Coronavirus disease-19 (COVID-19) is the disease caused by 2019-nCoV/SARS-CoV-2, a novel β coronavirus of group 2B [1]. The illness ranges from asymptomatic or mild infection to severe respiratory tract infections in humans such as those seen in severe acute respiratory syndrome (SARS) and Middle East respiratory syndrome (MERS). Presentations include fever, coughing, dyspnea, watery diarrhea, myalgia, severe lymphopenia, prolonged coagulation profiles, cardiac disease, and sudden death [2, 3].

Since the emergence in Wuhan, Hubei province, China in December 2019, COVID-19 has increased rapidly in China and progressed worldwide. On January 30, 2020, WHO declared the outbreak as a Public Health Emergency of International Concern (PHEIC). As of May 27, 5,488,825 cases have been confirmed globally, including in Americas, Europe, Eastern Mediterranean, South-East Asia, and Africa, and 349,095 deaths have been reported [4]. Coagulopathy was reported, and D-dimer elevations were seen in 3.75–68.0% of the COVID-19 patients [2, 5, 6]. Previous studies in community-acquired pneumonia (CAP) and chronic obstructive pulmonary disease (COPD) patients have shown that D-dimer level is higher in severe cases and may be used as a prognostic biomarker [7–9], and D-dimer > 1 μg/ml is one of the risk factors for mortality in adult inpatients with COVID-19 [6]. However, the role of D-dimer in COVID-19 patients has not been fully investigated. In this study, we showed D-dimer levels in patient groups stratified by clinical severities, imaging staging, in-hospital death, and assessed the role of D-dimer as a biomarker for disease severity and clinical outcome.

## Methods

### Patients

We enrolled patients of confirmed COVID-19 referred to the Renmin Hospital of Wuhan University (Wuhan, China), a designated center prioritized in treating critical illness, from January 28 to March 08, 2020. Confirmed cases were defined as those with epidemiological history, consistent with two clinical manifestations, and microbiological evidence (respiratory or blood specimens positive for SARS-CoV-2 by real-time reverse transcription polymerase chain reaction (RT-PCR) assay or virus gene sequencing) according to the Novel Coronavirus Pneumonia Diagnosis and Treatment Guideline (6th ed.) (in Chinese) published by the National Health Commission of China [10]. Symptom onset is determined by the earliest clinical manifestations consistent with COVID-19, such as fever, cough, dyspnea, muscle pain, diarrhea, and fatigue, recorded in medical history taken upon admission. Exclusion criteria included pregnancy, cancer, hematologic malignancy, chronic liver disease, acute coronary syndrome, surgery or trauma within 30 days, and patients without D-dimer testing upon admission. We retrospectively collected demographic, clinical data, laboratory parameters, chest CT imaging, and prognosis through electronic nursing and medical records using standardized data collection form. This study was approved by the institutional ethics board of Renmin Hospital of Wuhan University (No. WDRY2020-K048).

### Laboratory and imaging methods

Complete blood count, coagulation profile, renal and liver function, creatine kinase, electrolytes, myocardial enzymes, CD4 and CD8 cell counts, C-reactive protein, and procalcitonin were collected routinely on admission. D-dimer level is tested using immunoturbidimetric assay with reference range of 0–0.50 mg/L (Sysmex, CS5100). Doppler ultrasound and CT pulmonary angiography were done for any patients with high clinical suspicion of pulmonary embolism/deep vein thrombosis (PE/DVT). Chest CT scan was done for all inpatients.

### Severity assessment

Clinically, severity of the COVID-19 patients was classified into mild, moderate, severe, and critically ill according to the Novel Coronavirus Pneumonia Diagnosis and Treatment Guideline (6th ed.) by the National Health Commission of China (Supplement table 1) [10]. Radiologically, the area of affected lungs consistent with viral pneumonia in each patient’s first chest CT after admission was measured and classified into ≤ 30%, 31–50%, and ≥ 50% of total lung area. According to oxygenation index (OI) at admission, patients were grouped into 4 groups (group 1, OI ≥ 400 mmHg; group 2, OI 300–399 mmHg; group 3, OI 200–299 mmHg; group 4, OI < 200 mmHg). The scores of SOFA, qSOFA, ITSH for disseminated intravascular coagulation (DIC), CURB-65 for community-acquired pneumonia and Wells’ rule [11], and the revised Geneva score [12] for assessing pulmonary embolism (PE) risk for each patient were documented.

### Statistics

Continuous data accorded with normal distribution and homogeneity of variance were expressed as mean ± SD and compared by independent samples *t* test or expressed as median (25–75th percentile) and compared by Wilcoxon rank sum test. Categorical variables were expressed as number (percentage) and compared by Chi-square tests or Fisher’s exact test. To explore the risk factors associated with mortality, univariable and multivariable logistic regression models were used. Considering the total number of deaths (*n* = 17) in our study and to avoid overfitting in the model, we excluded variables from the univariable analysis if their between-group differences were not significant and if the number of events was too small to calculate odds ratios. Therefore, we chose age, SOFA, qSOFA, ISTH-DIC score, CURB-65, lymphocyte count, and D-dimer as the seven variables for our multivariable logistic regression model. Correlations of D-dimer with clinical staging, chest CT staging, oxygenation index, and in-hospital mortality were evaluated by Kendall’s tau-b coefficient analysis. To assess the predictive value of D-dimer for mortality, receiver operating characteristic (ROC) analysis was conducted with calculations of the area under the ROC curve (AUC), sensitivity and specificity. Statistical analyses were performed with SPSS (v.22.0; SPSS Inc., Chicago, IL, USA), and *P* value less than 0.05 was considered statistically significant.

## Results

As a designated referral center for the novel coronavirus infection, all the patients hospitalized were confirmed with RT-PCR. After excluding subjects using the exclusion criteria, we included 248 consecutive inpatients between January 28 and March 8, 2020, in the final analysis. The mean age of the 248 patients was 63.0 ± 13.4 years, ranging from 27 to 88 years. The average time from symptom onset to admission was 11.5 ± 5.1 days. Nearly one third of the patients had comorbidities, with hypertension being the most common (31.5%), followed by diabetes mellitus (17.7%). Mild to moderate cases, severe cases, and critically ill cases accounted for 36.3%, 43.5%, and 20.2% of the patients, respectively. Average length of hospital stay was 30.8 ± 12.4 days. Using the revised Geneva score, none belonged to the high probability group for risk of PE. Four patients belonged to the high probability group using the Wells’ rule. Fortunately, they were ruled out of PE/VTE by Doppler ultrasonography and CT pulmonary angiography. Seventeen patients died during hospitalization. D-dimer elevation (≥ 0.50 mg/L) was seen in 74.6% (185/248) of the patients.

Since all patients with normal D-dimer (< 0.5 mg/L) at admission survived, patients were grouped into D-dimer levels of < 1, 1–2, and > 2 mg/L in the univariable and multivariable logistic regression models (Table 1). In univariable analysis, age, SOFA score, qSOFA score, ISTH-DIC score, CURB-65, lymphocytopenia, and elevated D-dimer were associated with death. When these variables were included in the multivariable logistic regression model, D-dimer greater than 2 mg/L at admission was the only variable associated with increased odds of mortality [OR 10.17 (95% CI 1.10–94.38), *P* = 0.041].

**Table 1:** Risk factors associated with mortality among COVID-19 patients

	Univariable OR (95% CI)	*P* value	Multivariable OR (95% CI)	*P* value
**Age (years)**^a^	1.08 (1.03~1.14)	**0.004**	1.04 (0.98~1.10)	0.187
**Female (vs male)**	2.32 (0.83~6.48)	0.109	-	-
**Comorbidity present (*vs*not present)**				
**Hypertension**	1.58 (0.58~4.31)	0.374	-	-
**Diabetes mellitus**	2.83 (0.92~8.67)	0.069	-	-
**SOFA score**^a^	2.00 (1.50~2.69)	**0.000**	1.44 (0.89~2.33)	0.134
**qSOFA score**^a^	4.47 (2.22~8.99)	**0.000**	2.43 (0.87~6.77)	0.091
**ISTH-DIC score**				
**0–4**	1 (ref)	-		
**≥ 5**	24.43 (3.77~158.31)	**0.000**	1.40 (0.05~36.97)	0.84
**CURB-65 score**				
**0–1**	1 (ref)	**-**		
**2**	7.59 (2.20~26.25)	**0.001**	1.31 (0.26~6.71)	0.743
**≥ 3**	14.43 (3.89~53.56)	**0.000**	0.86 (0.09~8.05)	0.895
Lymphocyte count (10^9^/L)				
**< 1.0**	9.85 (2.54~37.80)	**0.001**	1.94 (0.24~15.57)	0.533
**≥ 1.0**	1 (ref)	-		
**Alanine aminotransferase (U/L)**				
**≤ 40 U/L**	1 (ref)	-	-	-
**> 40 U/L**	0.48 (0.13~1.72)	0.259	-	-
eGFR (ml/[min*1.73m^**2**^**])**				
**< 90**	1.67 (0.60~4.67)	0.326	-	-
**≥ 90**	1 (ref)	-	-	-
**Creatine kinase (U/L)**				
**≤ 198**	1 (ref)	-	-	-
**> 198**	2.18 (0.58~8.25)	0.250	-	-
**D-dimer (mg/L)**				
**< 1**	1 (ref)	-	1 (ref)	-
**1–2**	2.48 (0.15~40.47)	0.524	2.21 (0.12~38.61)	0.612
**> 2**	24.43 (3.16~189.00)	**0.002**	10.17 (1.10~94.38)	**0.041**
**Anticoagulation therapy**				
**No**	1 (ref)	**-**	-	
**Yes**	0.95(0.34~2.67)	**0.93**	-	

^a^ Per 1-unit increase

The comparison of demographic and clinical characteristics between the normal D-dimer group and elevated D-dimer group are shown in Table 2. Major laboratory markers and chest imaging features upon admission were recorded (Table 3). 35.5%, 31.0%, and 33.5% of the patients had affected lungs of ≤ 30%, 31–50%, and ≥ 50% of the total area. The predominant changes seen were ground glass opacity (54.0%), followed by patchy consolidation (21.4%), fibrous stripes (12.9%), and irregular consolidated nodules (11.7%). 67.7% of the patients received oxygen therapy, including nasal cannula/face mask (52.0%), non-invasive mechanical ventilation (10.5%), invasive mechanical ventilation (5.2%), and extracorporeal membrane oxygenation in one patient. Anticoagulation therapy was prescribed in 34.3% of the cases.

**Table 2:** Comparison of demographic and clinical characteristics between COVID-19 patients with normal and elevated D-dimers

	Normal D-dimer, *n* = 63	Elevated D-dimer, *n* = 185	*P* value
Age (years)	58.0 ± 14.4	64.6 ± 12.6	0.001
Male gender (%)	32 (50.8)	103 (55.7)	0.502
Underlying disease, n (%)			
Hypertension	12 (19.0)	66 (35.7)	0.014
Diabetes mellitus	7 (11.1)	37 (20.0)	0.319
Coronary artery disease	3 (4.8)	9 (4.9)	1.000
Chronic kidney disease	0 (0)	6 (3.2)	
Chronic obstructive pulmonary disease	0 (0)	4 (2.2)	
Time since symptom onset (days)	10.5 ± 4.8	11.8 ± 5.1	0.069
Highest temperature (°C)	38.1 ± 0.9	38.1 ± 1.0	0.948
Clinical staging at admission, *n* (%)			0.000
Mild-moderate	40 (63.5)	50 (27.0)	
Severe	20 (31.7)	88 (47.6)	
Critically ill	3 (4.8)	47 (25.4)	
Wells’ score, *n* (%)			0.230
< 2 points	60 (95.2)	167 (90.3)	
2–6 points	2 (3.2)	15 (8.1)	
> 6 points	1 (1.6)	3 (1.6)	
Geneva score, n (%)			0.105
0–3 points	34 (54.0)	78 (42.2)	
4–10 points	29 (46.0)	107 (57.8)	
≥ 11 points	0	0	
CURB-65, *n* (%)			0.008
Score 0	30 (47.62)	64 (34.59)	
Score 1	25 (39.68)	68 (36.76)	
Score 2	5 (7.94)	20 (10.81)	
Score 3	3 (4.76)	17 (9.19)	
Score 4	0 (0)	15 (8.11)	
Score 5	0 (0)	1 (0.54)	
SOFA score	1 (0–1)	1 (0–3)	0.007
qSOFA score	0 (0–0)	0 (0–1)	0.084
ITSH-DIC score	0 (0–0)	2 (2–3)	0.000
Oxygen treatment, *n* (%)			0.114
Nasal cannula/face mask	28 (44.4)	101 (54.6)	
NIMV	2 (3.2)	24 (13.0)	
IMV	0	12 (6.5)	
IMV + ECMO	0	1 (0.5)	
Anticoagulation therapy	12 (19.0)	73 (39.5)	0.000
Length of hospital stay	28.3 ± 12.5	31.7 ± 12.3	0.078
In-hospital mortality, *n* (%)	0	17 (9.2)	0.008

*NMV* non-invasive mechanical ventilation (including high flow nasal cannula), *INMV* invasive mechanical ventilation, *ECMO* extracorporeal membrane oxygenation

**Table 3:** Comparison of laboratory value and imaging characteristics between COVID-19 patients with normal and elevated D-dimers

	Normal D-dimer, *n* = 63	Elevated D-dimer, *n* = 185	*P* value
D-dimer (mg/L)	0.35 (0.23–0.42)	1.69 (0.91–5.06)	0.000
PaO_2_ (mm Hg)	71.63 ± 14.81	67.37 ± 14.48	0.147
PaCO_2_ (mm Hg)	42.5 ± 8.38	40.13 ± 7.07	0.112
White blood cell count (× 10^9^/L)	4.99 ± 2.44	6.71 ± 3.13	0.000
Lymphocyte count (× 10^9^/L)	1.3 ± 0.59	1.03 ± 0.60	0.002
Neutrophil count (× 10^9^/L)	3.12 ± 2.17	5.11 ± 3.13	0.000
Hemoglobin (g/L)	128.7 ± 13.3	122.6 ± 16.8	0.010
Platelet count (10^9^/L)	221.9 ± 82.9	232.9 ± 92.6	0.405
CD4 (cells/mm^3^)	337 (165.5–560.5)	263 (109.0–429.5)	0.326
CD8 (cells/mm^3^)	229 (92–367)	123 (48.25–226.25)	0.122
C-reactive protein (mg/L)	8.5 (5–35.85)	48.4 (10.98–92.25)	0.000
Procalcitonin (ng/mL)	0.05 (0.03–0.08)	0.08 (0.04–0.18)	0.000
Total bilirubin (μmol/L)	10.0 (6.7–13.0)	11.7 (8.6–15.5)	0.050
Combined bilirubin (μmol/L)	3.4 (2.6–5.0)	4.3 (3.3–6.1)	0.030
Alanine aminotransferase (U/L)	21.0 (13.0–39.0)	28.5 (19.0–55.3)	0.001
Aspartate aminotransferase (U/L)	24.0 (17.0–34.5)	33.5 (21.0–49.0)	0.001
Alkaline phosphatase (U/L)	60.4 ± 17.0	77.7 ± 40.3	0.000
Gamma-glutamyl Transferase (U/L)	30.0 (13.5–47.0)	33.5(23.0–68.3)	0.001
Lactate dehydrogenase (IU/L)	250.41 ± 90.4	356.11 ± 185.28	0.000
Serum creatinine (mmol/L)	64.0 (54.0–77.0)	64 (52.0–74.0)	0.715
eGFR (ml/[min*1.73m^2^])	97.15 ± 13.99	88.64 ± 24.94	0.001
Blood glucose (mmol/L)	6.49 ± 3.34	7.12 ± 3.94	0.257
Creatine kinase (U/L)	75.0 (50–132.5)	58.5 (33–87.5)	0.057
Prothrombin time (s)	11.7 (11.3–12.3)	12.0 (11.6–12.7)	0.005
Activated partial thromboplastin time (s)	28.2 (26.4–31.15)	27.3 (25.45–29.85)	0.016
Fibrinogen (g/L)	4.03 ± 1.33	4.80 ± 1.57	0.001
Cardiac troponin I (μg/L)	0.01 (0.01–0.01)	0.01 (0.01–0.02)	0.000
B-type natriuretic peptide (pg/mL)	44.45 (18.15–147.6)	222.25 (87.69–461.83)	0.000
Area of affected lung on Chest CT, *n* (%)			0.000
≤ 30%	37 (58.7)	51 (27.6)	
31–50%	16 (25.4)	61 (33.0)	
≥ 50%	10 (15.9)	73 (39.4)	
Predominant feature on Chest CT, *n* (%)			0.287
Ground glass opacities	40 (63.5)	94 (50.8)	
Patchy consolidations	12 (19.1)	41 (22.2)	
Fibrous stripes	4 (6.3)	25 (13.5)	
Irregular solid nodules	7 (11.1)	25 (13.5)	
Pericardial effusion, *n* (%)	1 (1.6)	4 (2.2)	0.800

**Fig. 1:**
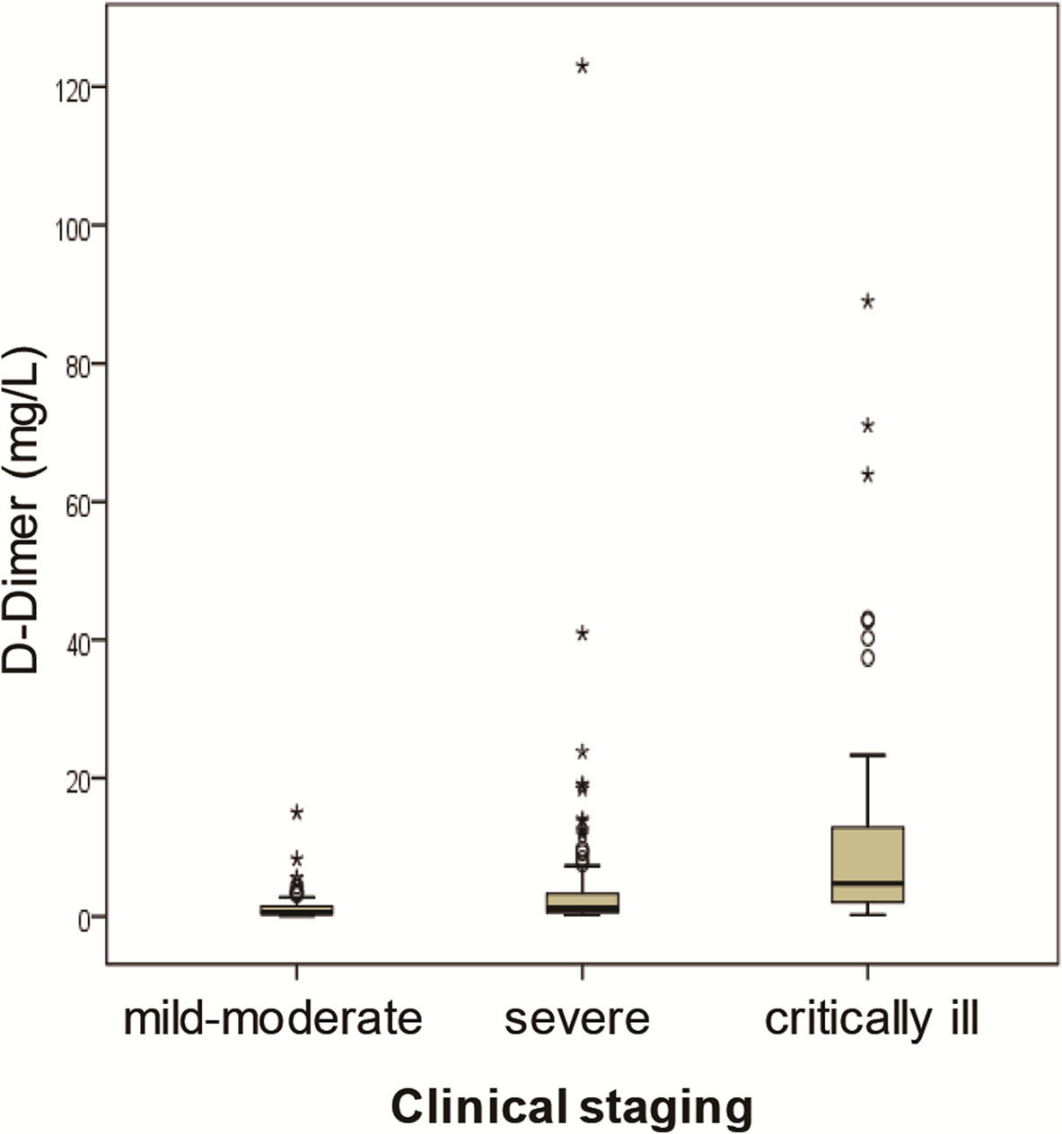
Correlations of D-dimer levels with clinical staging

**Fig. 2:**
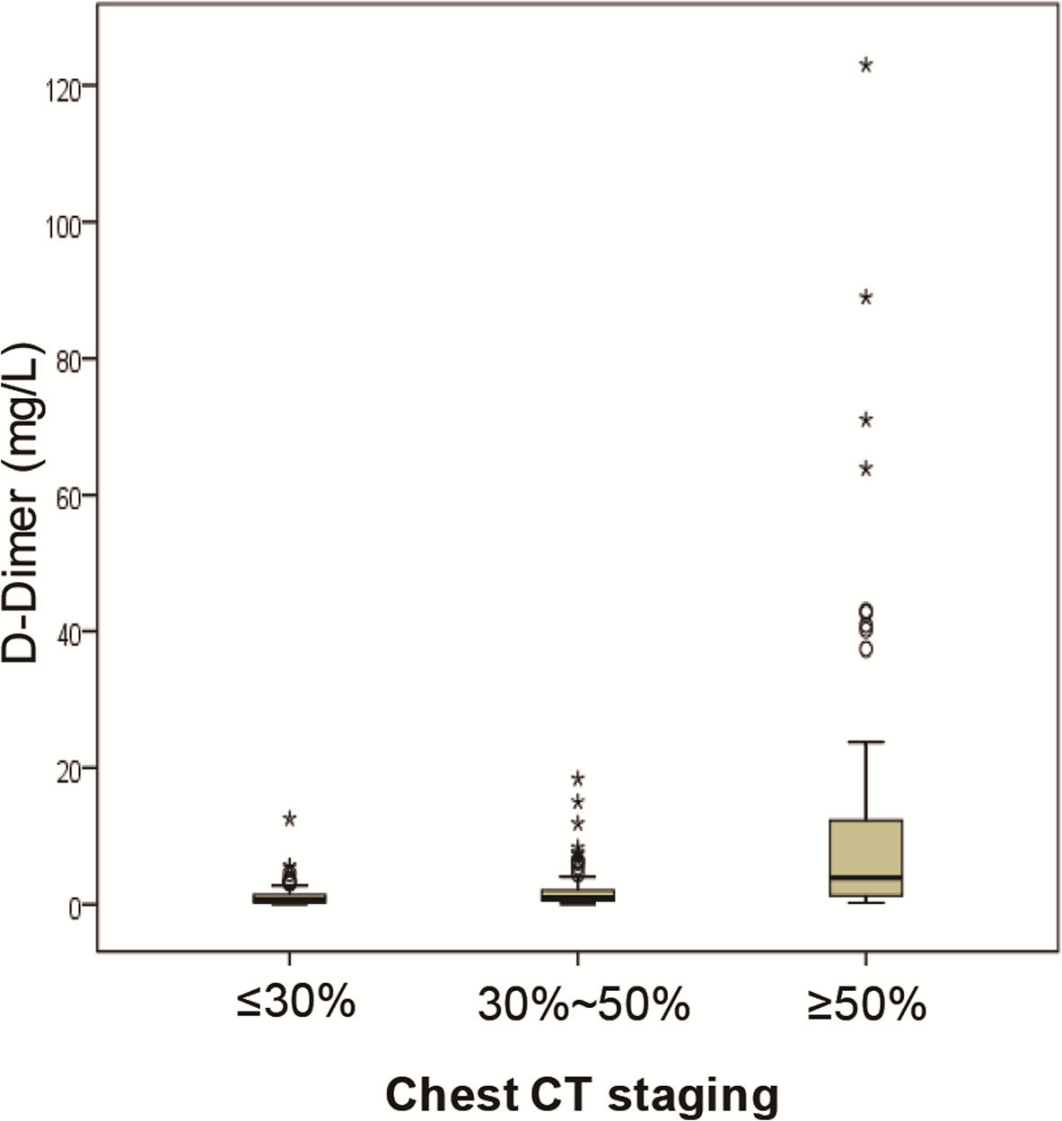
Correlations of D-dimer levels with chest CT staging according to area of affected lungs

**Fig. 3:**
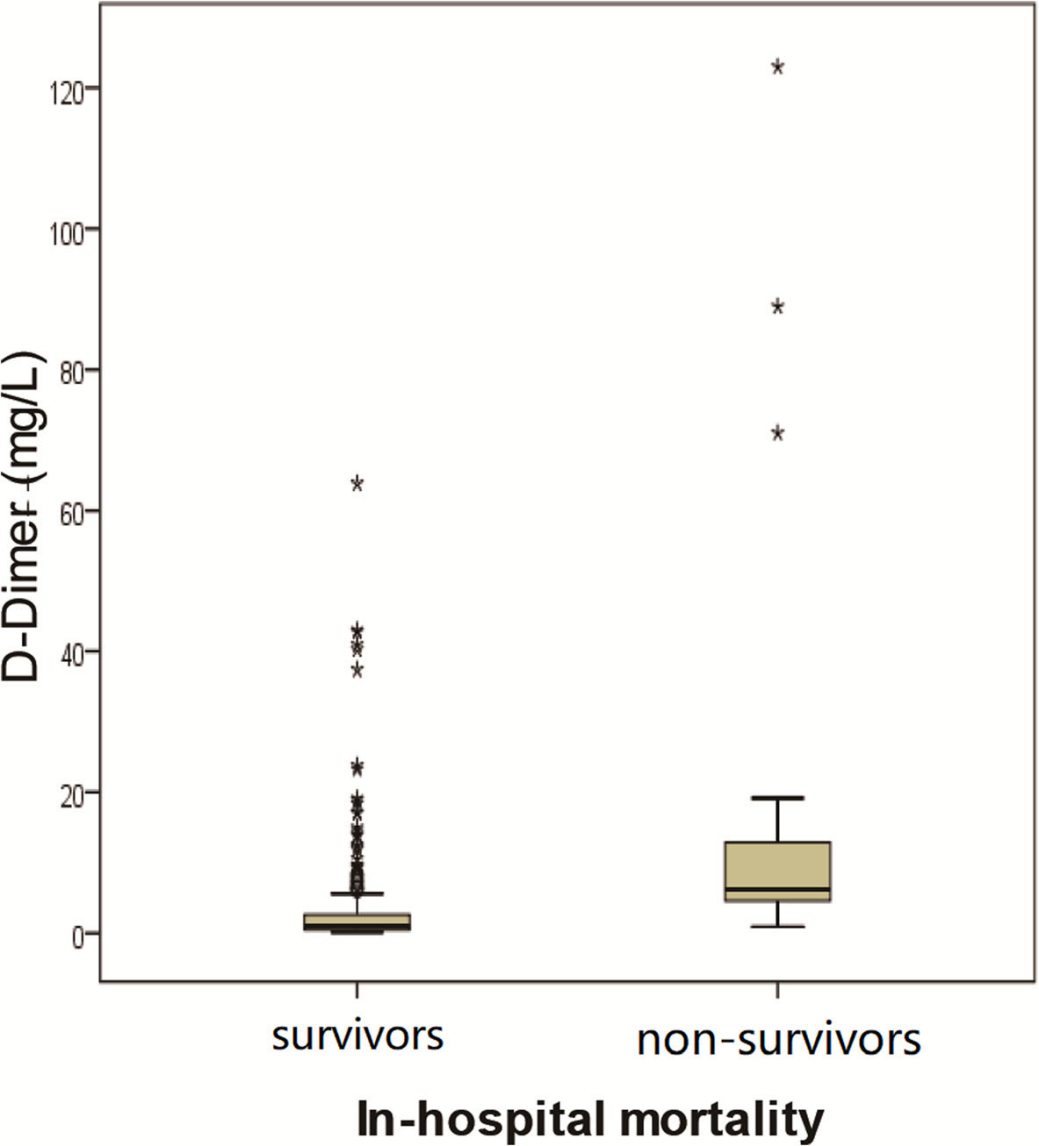
Correlations of D-dimer levels with in-hospital mortality

**Fig. 4:**
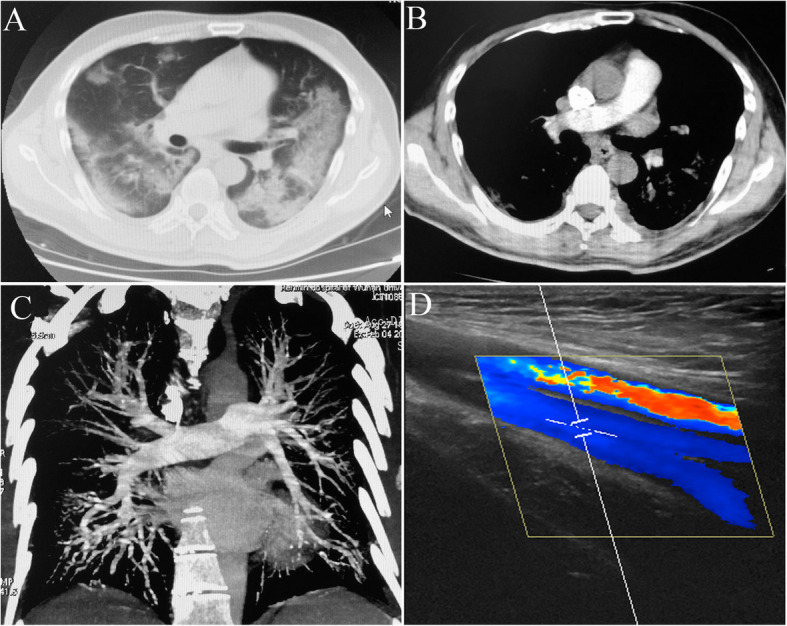
A 59-year-old male diagnosed with COVID-19 who presented with fever, coughing, and hemoptysis. Chest CT upon admission showing ground glass opacities and patchy consolidation (**a**). He had an elevated D-dimer level of 9.43 mg/L. Wells’ score, Geneva score, and CURB65 score were 7, 7, and 2 respectively. Wells’ score suggested high probability of pulmonary embolism. CT pulmonary angiography (**b**, **c**) and Doppler ultrasonography (**d**) were then carried out and ruled out pulmonary embolism and deep vein thrombosis in the lower extremities

The distributions of D-dimer levels among patients with different clinical staging, chest CT staging, and who survived and deceased during hospitalization are presented in Figs. 1, 2, 3, and 4. On admission, D-dimer levels significantly increased with increasing severity of COVID-19 as determined by clinical staging (Kendall’s tau-b = 0.374, *P* = 0.000), chest CT staging (Kendall’s tau-b = 0.378, *P* = 0.000), and oxygenation index (Kendall’s tau-b = 0.392, *P* = 0.000). Median D-dimer levels showed an about 7-fold increase from moderate to critically ill patients (4.76 [2.02–13.30] mg/L versus 0.6 [0.33–1.49] mg/L, *P* = 0.000), a 5-fold increase from patients with ≤ 30% affected lung area to ≥ 50% change (3.93 [1.28–12.31] mg/L versus 0.6 [0.33–1.42], *P* = 0.042), and an over 9-fold increase from oxygenation index groups 1 to 4 ( 6.17 [1.75–14.20] mg/L versus 0.64 [0.46–1.39] mg/L, *P* = 0.000). All of those who did not survive had increased D-dimer level upon admission. When compared between patients who survived and who died during hospitalization, a significantly higher D-dimer level was detected in non-survivors versus survivors (6.21 [3.79–16.01] mg/L versus 1.02 [0.47–2.66] mg/L, *P* = 0047).

**Fig. 5:**
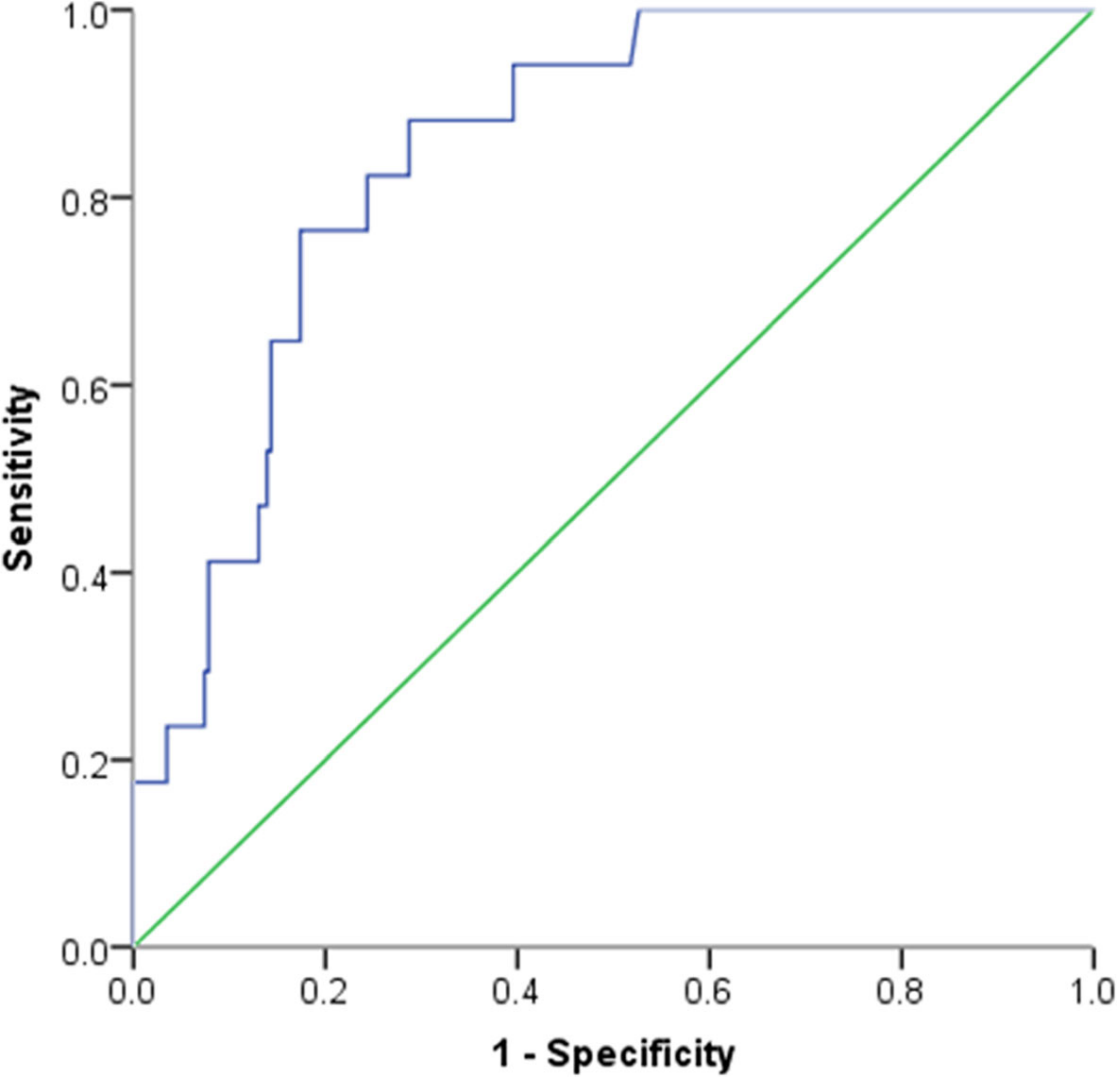
Receiver operating characteristic curve for D-dimer as parameter for predicting in-hospital mortality in COVID-19 patients

ROC analysis identified D-dimer > 2.14 mg/L upon admission as the optimal cutoff level to discriminate survivors from non-survivors (area under the ROC 0.85, standard error 0.037; 95% confidence interval [CI] 0.77–0.92, *P* = 0.000; Fig. 5). 32.7% of the included patients had a D-dimer of > 2.14 mg/L. For predicting in-hospital mortality, D-dimer level above 2.14 mg/L had a sensitivity of 88.2% and specificity of 71.3% (Table 4).

**Table 4:** Test characteristics of D-dimer for predicting in-hospital mortality with the optimal sensitivity and specificity scores

Cutoff point for D-dimer (mg/L)	2.14
Area under curve	0.85
95% CI	0.77–0.92
Subjects with D-dimer > 2.14 mg/L (%)	77 (31.2%)
Sensitivity (%)	88.2
Specificity (%)	71.3
Likelihood ratio	3.08

## Discussion

We demonstrated that in patients diagnosed with COVID-19, D-dimer elevation upon admission was common and was associated with both increased disease severity and in-hospital mortality. D-dimers are one of the fragments produced when plasmin cleaves fibrin to break down clots. The assays are routinely used as part of a diagnostic algorithm to exclude the diagnosis of thrombosis. However, any pathologic or non-pathologic process that increases fibrin production or breakdown also increases plasma D-dimer levels [13]. Examples include deep vein thrombosis/pulmonary embolism, arterial thrombosis, disseminated intravascular coagulation, and conditions such as pregnancy, inflammation, cancer, chronic liver diseases, post trauma and surgery status, and vasculitis. Among adults admitted to the emergency room, infections, instead of VTE/PE, are the most common reason for D-dimer elevation [14]. In the present study, no patient had confirmed PE/DVT, which supports the application of D-dimer in COVID-19 not just as a diagnostic tool for thromboembolism. In addition, only three patients in the elevated D-dimer group (3/185, 1.6%) with D-dimer levels of 42.8 mg/L, 89.0 mg/L, and 71.0 mg/L had ISTH-DIC scores of ≥ 5, which is laboratory evidence compatible with overt DIC. Thus, the majority of the included patients with D-dimer elevation in our study did not have overt DIC. Due to the retrospective nature of the study and small number of patients with ISTH-DIC score consistent with overt DIC, it is difficult to tell from our data if D-dimer elevation is related with DIC.

Several studies have shown that D-dimer levels are associated with severity of community-acquired pneumonia and clinical outcome [7, 15]. However, D-dimer has not been used as a biomarker for viral pneumonia [16, 17]. Though D-dimer elevation has been observed in articles describing the clinical features of COVID-19, whether the level of D-dimer is a marker of severity has not been examined.

In the present study, there is a significant correlation between D-dimer levels and disease severity stratified by the area of affected lungs on chest CT, oxygenation index, as well as clinical staging according to the interim guideline. In addition, a higher percentage of D-dimer elevation was seen in the present study than previously reported [2, 5]. This may be due to the higher percentage of severe/critically ill cases referred to our hospital, which is another demonstration of the correlation between D-dimer level and disease severity. This suggests that the assay may be used early as a marker of severity before chest CT scans or as a complement to CT and clinical staging.

In-hospital mortality was also associated with increased D-dimer levels, suggesting that the assay may be used as a single useful biomarker for clinical outcome in patients with COVID-19. Zhou et al. reported that D-dimer > 1 μg/ml is a risk for mortality [6]. The study objective, design, population, and statistical analysis of Zhou’s study and those of ours are different. Zhou’s study was a retrospective cohort study to describe risk factors for mortality and clinical course, which included patients who had been discharged or had died by January 31, 2020. The mortality rate was higher compared to that in our study (28.3% vs. 6.9%). To explore risk factor for mortality, Zhou et al. chose age, coronary heart disease, SOFA score, lymphocyte, and D-dimer as variables for multivariable logistic regression model. D-dimer was defined as a categorical variable in the analysis, and levels of ≤ 0.5 μg/L, > 0.5 to ≤ 1 μg/L, and > 1 μg/L were chosen. The laboratory method for D-dimer assay was not described. In the present case control study, we focused on the predictive value of D-dimer for in-hospital deaths using receiver operating characteristic analysis. In the analysis, D-dimer is defined as a continuous variable. Testing used immunoturbidimetric assay with reference range of 0–0.50 mg/L (Sysmex, CS5100). Despite the differences in study design and analysis, the findings and conclusions of the two studies are not inconsistent. Zhou et al. concluded that the potential risk factors of older age, high SOFA score, and D-dimer greater than 1 μg/L (instead of levels of ≤ 0.5 μg/L, or > 0.5 to ≤ 1 μg/L) could help clinicians to identify patients with poor prognosis. We found that when using the cutoff value of 2.14, D-dimer levels upon admission for in-hospital mortality has an AUC of 0.846. The sensitivity and specificity are 88.2% and 71.3%, respectively. The findings of this present study suggest that an elevated D-dimer level on admission (> 2.14 mg/L) may identify patients at higher risk for in-hospital mortality and therefore inform physicians about suitable candidates for intensive care and early intervention.

It is worth noting that the findings suggest associations between D-dimer levels and disease severity and mortality only. Evidence is still lacking as to the causal mechanisms and whether the associations are specific effects of SARS-CoV-2 infection or are consequences of systemic inflammatory response. In SARS-COV-2 infection, dysregulation of coagulation/anti-coagulation cascades results in worsening lung pathology [18]. In influenza, the pathogenesis by augmenting viral replication and immune pathogenesis can be attributed to an aberrant coagulation system, including both the cellular and protein components [19]. The pathological features of COVID-19 include diffuse alveolar damage with cellular fibromyxoid exudates, desquamation of pneumocytes and hyaline membrane formation, pulmonary edema with hyaline membrane formation, and interstitial mononuclear inflammatory infiltrates, dominated by lymphocytes, which greatly resemble those seen in SARS and MERS coronavirus infection [20, 21]. Presumably, the observed D-dimer elevation signify a hyperfibrinolysis state and increased inflammatory burden induced in SARS-COV-2 infection. In our logistic regression model to estimate risk factors associated with mortality, systematic anticoagulation therapy was not significantly associated with reduced risk of mortality. However, in a recent observational study including 2773 hospitalized COVID-19 patients, Paranjpe et al*.* found that treatment dose anticoagulant was associated with a reduced risk of mortality, especially among patients who required mechanical ventilation [22]. And longer duration of treatment was associated with a reduced risk of mortality (adjusted HR of 0.86 per day, 95% CI 0.82–0.89, *p* < 0.001). Whether anticoagulation therapy confers a survival benefit in patients hospitalized for COVID-19 needs further research with prospective randomized trials. Currently, the potential benefits need to be weighed against risk of bleeding.

This study has some limitations. First, the current study was done in a single center. The overall mortality (6.9%) was lower compared with that reported in other studies done in Wuhan [2, 6] and considerably higher than those reported by other provinces [5, 23]. Further researches may be needed when extrapolated to wider patient population. Second, the study is retrospective in nature. The patients included were not systematically assessed for the presence of PE/DVT but only when clinically suspected. Third, we did not look into the value of serial D-dimer monitoring in assessing COVID-19 patients.

## Conclusions

In conclusion, D-dimer levels are commonly elevated in patients infected with SARS-CoV-2. Significantly higher levels are found in those with critical illness and may be used as a prognostic marker for in-hospital mortality.

## Supplementary information

**Additional file 1: Supplement table 1.** Clinical Classifications of COVID-19.

## Data Availability

The dataset supporting the conclusions of this article is included within the article and its additional files.

## Abbreviations

COVID-19: Coronavirus disease-19
SARS: Severe acute respiratory syndrome
MERS: Middle East respiratory syndrome
PHEIC: Public Health Emergency of International Concern
CAP: Community-acquired pneumonia
COPD: Chronic obstructive pulmonary disease
PE: Pulmonary embolism
DVT: Deep vein thrombosis
ISTH: International Society on Thrombosis and Haemostasis
DIC: Disseminated intravascular coagulation
