# Supplementary material for: D-dimer as a biomarker for disease severity and mortality in COVID-19 patients: a case control study
Source: J Intensive Care. 2020 Jul 10;8:49. doi: 10.1186/s40560-020-00466-z (PMC7348129; doi:10.1186/s40560-020-00466-z)
Supplement: Supplementary file 1 — Additional file 1: Supplement table 1. Clinical Classifications of COVID-19. [file 40560_2020_466_MOESM1_ESM.docx]

**Supplement table 1.**

**Clinical Classifications of COVID-19**

| 1.Mild Cases |
| --- |
| The clinical symptoms are mild and no pneumonia can be found in imaging. |
| 2.Moderate Cases |
| Patients have symptoms such as fever and respiratory tract symptom. Pneumonia can be seen in imaging. |
| 3.Severe Cases |
| Meeting any of the following:   - Respiratory distress, RR >30breaths/min; - Oxygen saturation less than 93% at resting state; - Arterial partial pressure of oxygen (PaO2/ oxygen concentration (FiO2) <300 mmHg (1 mmHg=0.133 kPa). - Patients with >50% lesions progression within 24 to 48 hours in chest imaging should be managed as severe cases. |
| 4.Critically ill cases |
| Meeting any of the following:   - Respiratory failure occurs and mechanical ventilation is required; - Shock occurs; - Complicated with other organ failure that requires monitoring and treatment in ICU. |
